# Super Hydrophobic SiO_2_/Phenolic Resin-Coated Filter Screen and Its Application in Efficient Oil–Water Separation

**DOI:** 10.3390/ma15238395

**Published:** 2022-11-25

**Authors:** Yan Zhao, Zhongmin Xiao, Ziming Feng, Qing Luo, Xiaoping Liu, Wei Cui

**Affiliations:** 1 School of Mechanical Science and Engineering, Northeast Petroleum University, Daqing 163318, China; 2 School of Mechanical and Aerospace Engineering, Nanyang Technological University, 50 Nanyang Avenue, Singapore 639798, Singapore; 3 College of Mechanical and Electrical Engineering, Wenzhou University, Wenzhou 325000, China; 4 Business Development Department, Daqing Oilfield Co., Ltd., Daqing 163000, China; 5 Institute of Geotechnology, Daqing Yushulin Oilfield Development Co., Ltd., Daqing 163000, China; 6 School of Energy and Power Engineering, University of Shanghai for Science and Technology, Shanghai 200093, China

**Keywords:** hydrophobic nano-SiO_2_, super hydrophobic coating film, phenolic resin, filter screen, oil-water separation

## Abstract

The discharge of industrial liquid waste continues to cause more and more environmental problems. The current research aims at developing a durable and highly efficient filter screen for oil-water separation. In this paper, hydrophobic nano-SiO_2_ and phenolic resin were used as raw materials. Hydrophobic SiO_2_ particles were fixed on the surface of the coated filter screen by heating and curing the anchored particles. The surface morphology, element composition, surface roughness and water contact angle of the prepared super hydrophobic SiO_2_/phenolic resin-coated filter screen were analyzed and discussed by using SEM, EDS, AFM, OCA and other instruments. The results showed that the prepared filter screen contained Si, O, C elements, which proved that the resin coating film had adhered to the filter screen surface. When the aperture of the phenolic resin-coated filter screen was 400 meshes, the drainage angle reached a maximum value of 153.8° ± 0.8°. When two layers of hydrophobic SiO_2_ phenolic resin were coated on the screen, the surface of the filter screen had a sufficient nano-porous structure and high roughness. The tests showed that the minimum water contact angle of the filter screen exceeded 150°, which indicated excellent chemical resistance. Through the analysis of oil-water separation efficiency of isooctane, gasoline, n-hexane, dodecane, edible oil, dichloromethane and trichloromethane, it was concluded that the lowest separation efficiency for edible oil was 97.2%, and the highest separation efficiency for n-hexane was 99.4%. After 50 cycles of separation, the oil-water separation efficiency for n-hexane was still at 99%.

## 1. Introduction

Petroleum production, transportation and usage inevitably lead to oil leakage, which seriously threatens the ecological environment. How to treat oily sewage is the key to protecting the environment. Oil–water separation technology is based on the different physical and chemical properties of oil and water, such as gravity separation technology, filtration, centrifugation, and electrochemical methods [1]. However, the separation efficiency of these conventional oil-water separation technologies is extremely low, and it is difficult to recover oil. There is a high demand in finding simpler and more efficient oil-water separation technologies. Researchers have tried to adjust the inherent wettability of the interface to improve the flow behavior of the liquid phase on the solid surface. Another possible solution is to change the resistance of the filter medium to oil and water.

Recently, the application of superhydrophobic surface preparation has attracted more and more attention, especially in the research process. Liu [2] prepared a super hydrophilic underwater and super oil-repellent mesh membrane by a two-step hydrothermal method to coat pure inorganic ZnO-Co_3_O_4_ onto the surface of copper mesh. The contact angle of the oil under water could reach 159.2° ± 1.3° (with dichloroethane as the oil drop). Using a polyester non-woven fabric as the matrix and polyphenyloxazine (PBZ) and TiO_2_ as the raw materials, Xin [3] developed a simple dip coating and thermal curing method to construct the polyphenyloxazine/TiO_2_-modified fabric, which has super hydrophobic and super lipophilic properties. It can not only efficiently separate the oil-water mixture, but it can also decompose methylene blue under ultraviolet light to achieve self-cleaning of the membrane surface. In Gondal et al. [4], the stainless steel mesh surface was coated with a WO_3_ nano-structure via the spraying method to prepare the super hydrophilic underwater super oil-repellent composite mesh membrane. Under the self-gravity drive, the oil-water separation efficiency was significantly improved. Naoyuki Yokoi [5] prepared a super hydrophobic and super lipophilic mesh membrane material via the simple spraying method using alkali-treated polyester mesh as the matrix and SiO_2_ and perfluorooctyl triethoxysilane as the raw materials. The produced membrane had high wear resistance, acid and alkali resistance (pH = 2–14), and excellent oil-water separation performance.

SiO_2_ nanoparticles have attracted extensive research due to their low density, high porosity, low thermal conductivity, and other advantages. Yanbao Guo [6] prepared lipophilic and hydrophobic silica sol via a sol–gel method and dipped lipophilic and hydrophobic coating on a stainless steel net. Separation experiments of various oil-water mixtures were carried out. They found that the modified mesh had better lipophilic and hydrophobic properties. Consistent lipophilicity and hydrophobicity were obtained under ultrasonic cleaning conditions and in different pH (3–11) solutions. The modified filter screen had good separation and reusability for different types of oil-water mixtures. Song [7] proposed a new method to construct a three-dimensional hydrophobic nano-SiO_2_ porous TIM(thermal insulating material) through a micro-lotion treatment. The polymethyl-hydro-siloxane-modified TIM had a large water contact angle of 166° and had excellent durability under high temperatures up to 400 °C, 100% high humidity and chemical attack. Liu [8] prepared SiO_2_ nanospheres with surface mercaptan groups by using 3-mercaptopropyltriethoxysilane (KH590) and hydroxyl condensation reaction on the surface of silicon (SiO_2_) particles. This material effectively absorbed more than four times the oil solvent, and the separation efficiency was more than 99%. Under strong acid and alkali conditions or a seawater environment, SiO_2_ nanospheres also have more than 20 times the effective oil absorption capacity and high recoverability. To improve the thermal stability and waterproof performance of ordinary cotton, Xu [9] added hydrophobic SiO_2_ particles, WPUA (waterborne polyurethane acrylate) and silica aerogel (SA) powder onto the surface of the coating film. The experimental results showed that the thermal stability of coated fabrics is significantly improved by adding aeroneneneba gel and silane-modified SiO_2_. The water contact angle of the film increased significantly when the silane-modified SiO_2_ particles were mixed with SA/WPUA. Liu [10] combined a poly (methyl-3,3,3-trifluoropropyl siloxane) (PMTFPS)-modified silicon with low surface free energy fluorosilicone resin to design an ice-resistant surface with super hydrophobicity. The contact angle and rolling angle of the SiO_2_-PMTFPS coating reached 158.5° and 1°, respectively. After immersion in solutions of different pH and temperature, the coating had good chemical durability in an aqueous solution.

Metal porous mesh membranes (such as stainless steel mesh, copper mesh, nickel mesh, etc.) have the advantages of good flexibility, strong pressure resistance, easy surface processing, etc., and are often used as the matrix of special wetting oil-water separation membrane materials. Extensive research has been carried out to construct micro-nano-rough structures, as well as the modification of hydrophilic substances on the surface of a metal mesh membrane. Lei [11] first developed a polytetrafluoroethylene coating on the surface of the stainless steel mesh, prepared a super hydrophobic and super lipophilic stainless steel mesh film material, and applied it to the study of oil-water separation. Tian [12] prepared a vertical array of micro/nano-hierarchical zinc oxide nanorods on the surface of stainless steel mesh by two solution deposition methods, obtained a super hydrophilic, underwater super hydrophobic film, and analyzed the oil-water separation mechanism in detail. Zhang [13] constructed a new type of super hydrophilic, underwater super oleophobic inorganic membrane material. The membrane was made of Cu(OH)_2_ nanowires grown on a copper mesh, which can effectively separate immiscible oil/water mixture and oil in water lotion by gravity. The membrane with high separation flux did not reduce the flux for the continuous separation of 10 L of oil/water mixture. It also had excellent antifouling performance, good oil-water separation effect, and low synthesis cost. Raturi [14] prepared zinc oxide nanowires (NWs), which were coated on a stainless steel (SS) grid by chemical vapor deposition. The synthesized ZnO nws coating mesh showed super underwater hydrophilic, super hydrophobic behavior. This mesh worked in “dehydration” mode, and its super hydrophilic and underwater super hydrophobic properties allowed water to penetrate through the mesh easily while preventing oil passing. The wettability of the Z-nws coating mesh can easily change from a super hydrophilic state to super hydrophobic state by alternating annealing at 300 °C, and vice versa in a hydrogen and oxygen environment.

In summary, people began to study the surface modification of the filter screen, build nanostructures on the surface of the filter screen, and combine them with micron-sized meshes to give the filter screen super hydrophobic/super lipophilic properties. Super hydrophobic/super lipophilic materials are characterized by hydrophobic and lipophilic properties, which can effectively achieve oil-water separation, and then recover the oil. At present, a lot of effort is put into developing various oil-water separation materials with special wettability and super hydrophobicity to achieve the purpose of greatly improving oil-water separation efficiency. New technologies have been explored that can successfully prepare materials for oil-water separation, but these technologies still have many limitations in the oil-water separation process, such as complex preparation process and harsh preparation conditions. Some technologies can only be applied to special substrates, and some still need to use strong acid or strong alkaline reagents, which pollute the environment. The prepared oil-water separation materials have the disadvantages of poor stability and cannot be used for a long time. It is of great significance to rapidly produce stable and repeatable super hydrophobic and super lipophilic mesh membranes from common cheap raw materials and simple preparation methods. 

Because the separation efficiency of traditional oil-water separation materials is not high, the surface roughness of a traditional phenolic resin-coated filter screen is limited, resulting in insufficient hydrophobicity, which reduces the oil-water separation efficiency of the filter screen. In this paper, hydrophobic silicon dioxide particles are introduced into the phenolic resin system by curing method, and the SiO_2_ nanoparticles/phenolic resin coating film is prepared on the surface of the filter screen. Compared with the traditional phenolic resin coating film, due to the introduction of hydrophobic SiO_2_ nano-examples, the phenolic resin-coated filter screen increases the surface roughness of the filter screen, improves the surface hydrophobicity of the filter screen, improves the chemical resistance of the coating film, and improves the oil-water separation efficiency.

Hydrophobic SiO_2_ nanoparticles are widely used in many industrial fields due to their simple preparation, excellent performance, and easy access. Stainless steel filters have excellent mechanical, chemical and high-temperature resistance. In our current study, hydrophobic SiO_2_ nanoparticles were heated and solidified on a stainless steel filter screen through phenolic resin. Experimental and analyzing equipment such as SEM (scanning electron microscope), EDS (energy dispersion spectrum), OCA (overview contact angle), etc., were employed to investigate the surface morphology, element composition, water contact angle and other parameters of a SiO_2_ nano-phenolic resin filter screen. Six oil-water mixtures were used to test the oil-water separation performance of the filter screen. Obtained results were compared with those of relevant researchers. It was found that the developed filter screen has significant advantages in oil-water separation efficiency.

## 2. Materials and Methods

### 2.1. Research Route

**Figure 1:**
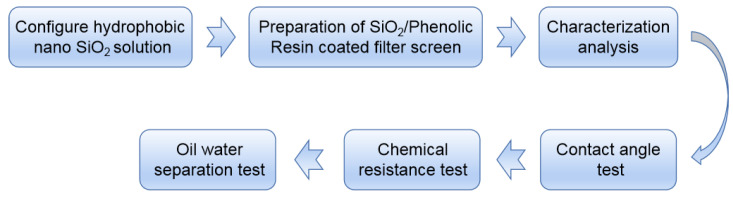
Test flow chart.

The test process is shown in Figure 1. Firstly, hydrophobic nano-SiO_2_ and hydrophobic SiO_2_ solutions were developed. Then, the SiO_2_/phenolic resin-coated filter screen was prepared with different concentrations of SiO_2_ solution. The state of hydrophobic nano-SiO_2_ particles was observed by transmission electron microscope at different temperatures. The scanning electron microscope and atomic force microscope were used to observe the surface morphology of hydrophobic SiO_2_/phenolic resin-coated filter screen. EDS was used to test the element composition of the filter screen. OCA method was employed to test the rolling angle of the filter screen surface. HCL solution, NaOH solution and other reagents were used to test the chemical resistance of the filter screen. Finally, six different oil-water mixtures were used to test the oil-water separation efficiency of the filter screen.

### 2.2. Experimental Materials and Instruments

The main drugs and reagents required for the test are shown in Table 1. The main drugs were purchased from manufacturers, and their specifications were also tested by manufacturers. The main test equipment and instruments required for the test are shown in Table 2.

### 2.3. Preparation of the SiO_2_/Phenolic Resin Filter Screen

A certain amount of phenolic resin was weighed and dissolved in ethyl acetate solvent using intelligent digital ultrasonic instrument for 10 to 30 min to prepare the 5% phenolic resin solution. A certain amount of hydrophobic SiO_2_ was weighed and dispersed in the solvent. It was dissolved in ultrasound for 10 to 30 min to prepare hydrophobic SiO_2_ dispersions of different concentrations. Spin coating phenol formaldehyde resin ethyl acetate solution and hydrophobic SiO_2_ ethyl acetate solution on glass slide and filter screen were processed with a desktop homogenizer. After solidifying them in an electric thermostatic blast drying oven at 160 °C for 30 min, a super hydrophobic film and film filter screen were developed.

### 2.4. Characterization Analysis

The morphologies of membrane surface were observed with the SEM (SEM, Quanta FEI 450, Boynton Beach, FL, USA) after being coated with hydrophobic nano-SiO_2_. An atomic force microscope (AFM, Agilent-S5500, Agilent, Palo Alto, CA, USA) was employed to analyze the surface states of the prepared membranes. The structure and morphology of hydrophobic nano-SiO_2_ were analyzed with the X-ray diffractometer (XRD, D8 Advance, Bruker, Billerica, MA, USA) CuK α Ray (λ = 1.5418 Å); The particle size distribution of hydrophobic nano-SiO_2_ was observed by the transmission electron microscopy (TEM, JEM-2100, Akishima, Japan). Thermogravimetric analysis (TGA/SDTA851) was conducted to test the heat resistance of hydrophobic nano-SiO_2_. The surface roughness was tested by the atomic force microscope (AFM, Nanoscope IIIa Multimode, Santa Barbara, CA, USA).

### 2.5. Contact Angle Test

The contact angle, rolling angle and oil contact angle of deionized water on the film surface were measured using the contact angle measuring instrument of POWEREACH of Shanghai Zhongchen Digital Technology Equipment Co., Ltd. (Shanghai, China).

Specific process: The sample was placed on the platform of the contact angle measuring instrument, and a micro syringe was used to take 5 μL of deionized water (or isooctane) to drop it on the sample surface. The contact angle and the rolling angle were measured. The method of measuring the oil contact angle was the same as that for water contact angle.

### 2.6. Chemical Resistance Test

To evaluate the performance of samples under extreme pH conditions, the following experiments were conducted to test the chemical resistance of a hydrophobic SiO_2_/phenolic resin-coated filter screen.

The three filter screen samples were soaked in three solutions, respectively, sealed and left to stand. The contact angle of the sample was measured at intervals. The preparation of solution: 0.098 g of 37% HCl, was weighed and dissolved in 100 mL of deionized water to obtain an acid solution with pH = 2; then, 0.04 g of NaOH was weighed and dissolved into 100 mL of deionized water to obtain alkaline solution with pH = 13; 5 g NaCl was weighed and dissolved into 100 mL deionized water to obtain 3.5 wt.% salt solution with pH = 7. The filter screen was soaked in toluene, ethyl acetate, acetone, tetrahydrofuran, and ethanol for a certain time, and the change of water contact angle was tested on the surface of the screen before and after soaking.

### 2.7. Oil–Water Separation Test

Preparation process of SiO_2_/phenolic resin-coated filter screen: A small amount of Sudan II was used to dye isooctane and other oils. A small amount of methylene blue was used to dye deionized water for experimental observation. Oil–water separation device: the prepared sample was cut into a 3 × 3 cm square, placed between two clamps, and the sample was clamped and sealed. The upper and lower ends of the clamps were connected with quartz glass tubes, and the device was placed at an angle of 45°. Oil–water separation test: an appropriate amount (about 15 g) of deionized water and appropriate amount of oil were weighed with a balance, mixed and then poured into the oil-water separation device. The super hydrophobic and super lipophilic filter screen sample can support the deionized water, but the oil passes through the screen. The trapped deionized water was weighed with a balance, and the mass of this part of deionized water was denoted by m_0_. Then, the separation efficiency of the filter screen sample is [15]:

(1) $$\eta =\frac{m_{0}}{m}\times 100\%$$

where *m* is the quality of deionized water.

### 2.8. Characterization of Hydrophobic SiO_2_

**Figure 2:**
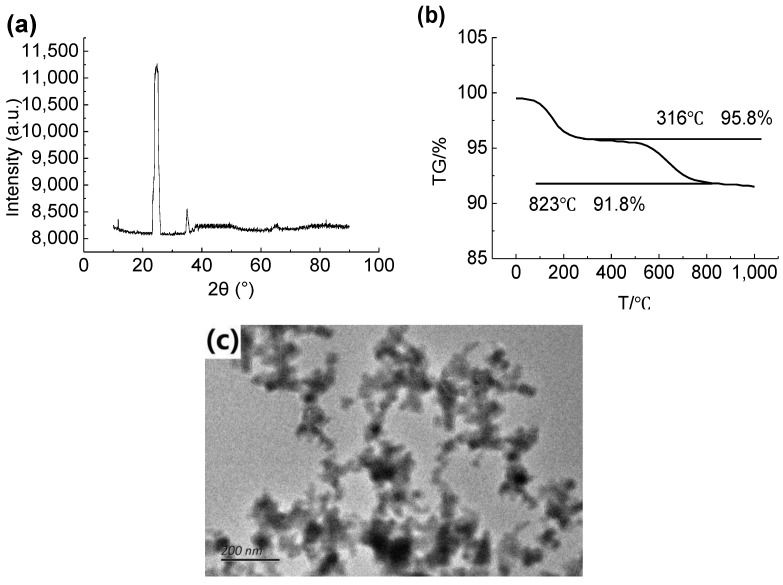
(**a**) Spectra of SiO_2_, (**b**) TG and (**c**) TEM images of SiO_2_.

Hydrophobic nano-SiO_2_ was used to construct the surface structure of superhydrophobic coating film. It can be seen from Figure 2a that the morphology of hydrophobic nano-SiO_2_ used was amorphous. Figure 2b shows that SiO_2_ nanoparticles have good heat resistance. Its weight lost 4.2% at 316 °C because of the adsorption of a few other substances on the surface. However, between 316 and 823 °C, due to the partial decomposition of hydrophobic nano-SiO_2_, about 8.2% of the mass percentage was lost [16]. The transmission electron microscope image of hydrophobic nano-SiO2 is shown in Figure 2c. The dark part in the figure shows SiO_2_ nano-particles, but the dark degree is different at different positions. Because the particles reach the nano-meter level, a large number of positive and negative charges accumulate on their surface area. The agglomeration of surface charges causes the agglomeration of nano-particles, and the van der Waals force between them was far greater than their own gravity, such that a large number of particles were concentrated together to form an agglomeration phenomenon. However, the agglomeration of SiO_2_ particles affects the coating filter screen. Before the test, ultrasonic dispersion is required to inhibit the agglomeration of SiO_2_ nanoparticles, which is relatively dispersed under TEM. It was observed that the SiO_2_ cluster composed of SiO_2_ has a diameter of tens of nanometers. It can be considered that the agglomeration of SiO_2_ was effectively suppressed; SiO_2_ can be used in the next experiment.

### 2.9. Contact Angle Analysis of the SiO_2_/Phenolic Resin-Coated Filter Screen

**Figure 3:**
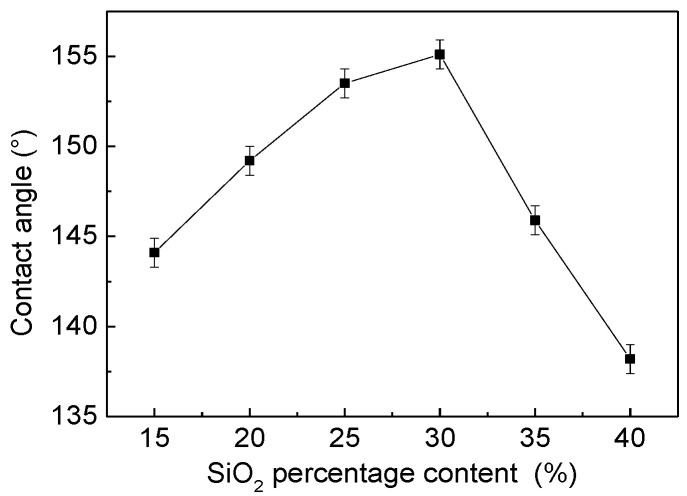
The relationship between the mass of SiO_2_ and water contact angles. The phenolic resin amount was 5%.

As shown in Figure 3, the mass percentage of hydrophobic nano-SiO_2_ was controlled to change from 15% to 40% with the filter screen as the base material. It was found that the water contact angle was gradually increased at first, and then decreased after reaching the highest point. When the content of hydrophobic nano-SiO_2_ increases from 15% to 30%, the water contact angle increases from 144.1° ± 0.8° to 153.5° ± 1.2° and then to 155.1° ± 1.3°, continuously. When the amount of SiO_2_ was 40%, the water contact angle decreased from 155.1° ± 1.3° to 145.9° ± 0.9°, and to 138.2° ± 0.8°, finally.

## 3. Results and Discussion

### 3.1. Analysis of Chemical Components on the Surface of the SiO_2_/Phenolic Resin-Coated Filter Screen

A superhydrophobic SiO_2_/phenolic resin-coated filter screen was obtained by curing SiO_2_ nanoparticles on the surface of a phenolic resin-coated filter screen in order to detect the curing reaction effect of phenolic resin. In this paper, we used infrared spectroscopy to test the spectra of phenolic resin and EDS to detect the element composition of the coated filter screen to confirm the presence of SiO_2_ particles in the coating film.

**Figure 4:**
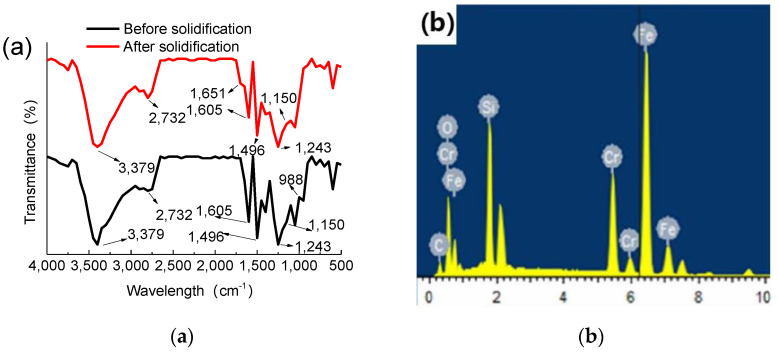
(**a**) Infrared spectra of phenolic resin before and after curing at 160 °C. (**b**) Element content of superhydrophobic and superhydrophobic SiO_2_/phenolic resin filter film.

Figure 4a shows the infrared spectrum of phenolic resin before and after curing at 160 °C. Figure 4b shows the element content of the super hydrophobic SiO_2_/phenolic resin film filter screen. From Figure 4a, by comparing the infrared spectra of phenolic resin before and after curing, the strong and wide absorption band of 3379 cm^−1^ of phenolic resin before and after thermal curing is the absorption peak of the -OH bond of phenol. The C-H stretching vibration absorption peak of methylene near 2732 cm^−1^ is not obvious. Furthermore, 1605 and 1496 cm^−1^ are the vibration absorption peaks of the benzene ring skeleton stretching, 1243 cm^−1^ is the phenol hydroxyl stretching vibration absorption peak, and 1150 cm^−1^ is the ether bond stretching vibration absorption peak. The shoulder peak appearing at 1651 cm^−1^ after curing is the carbonyl C=O stretching vibration absorption peak. The hydroxymethyl C-O is formed by curing reaction. The stretching vibration disappears at 988 cm^−1^, indicating that the phenolic resin undergoes a crosslinking reaction under the curing conditions [17]. It can be seen from Figure 4b that in addition to its own elements such as Fe, Cr, Ni and Mn, the stainless-steel filter screen also contains the elements C, O and Si. The elements C and O come from the phenolic resin, and the element Si comes from the hydrophobic SiO_2_ particles. The appearance of Si, O and C elements indicates that the spin-coated SiO_2_ particles and phenolic resin have successfully adhered to the filter screen.

### 3.2. Influence of Filter Screens with Different Apertures on Contact Angle and Rolling Angle of Coating Film

**Figure 5:**
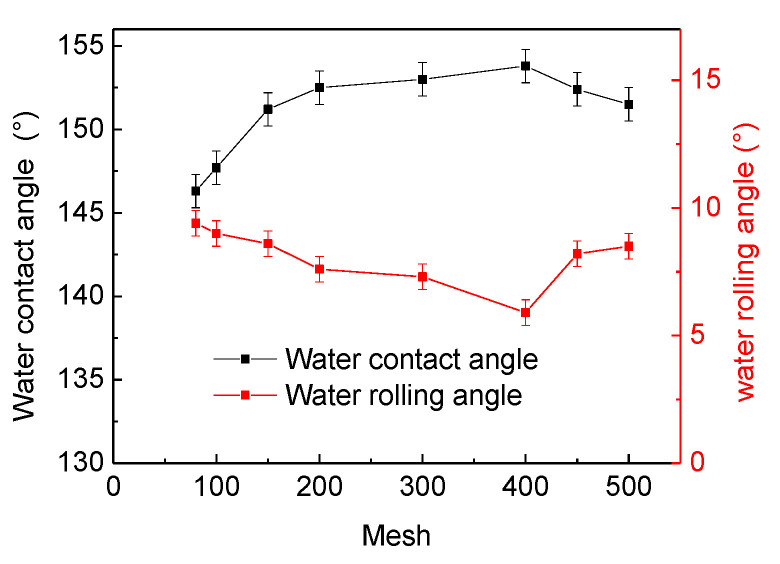
Trend chart for the influence of different aperture filters on contact angle and rolling angle of coatings.

Different filter screen apertures will affect the hydrophobicity of the SiO_2_/phenolic resin-coated filter screen. In order to study the relationship between the filter screen aperture and the hydrophobicity of the coated filter screen, we used filters with different apertures for the hydrophobic angle test in this paper, and the test results are shown in Figure 5.

Figure 5 depicts the trend chart for the influence of filter screens with different apertures on the contact angle and rolling angle of the coating film. We explored the influence of filter screen apertures of 500, 450, 400, 300, 200, 150, 100 and 80 mesh on the water contact angle and rolling angle. It can be seen from Figure 5 that the water contact angle on the film filter screen is rising and the rolling angle is declining when the SiO_2_ is 30%, the phenolic resin is 0.5%, and the aperture is between 100 and 400 mesh. When the aperture is 400 mesh, the maximum water contact angle of the film filter screen is 153.8° ± 0.8°. When the aperture exceeds 400 mesh, the water contact angle starts to decline, and the rolling angle shows an upward trend. The possible reason is that it is difficult for the coating film to capture the air through its surface structure, thus leading to a decrease in hydrophobicity when the pore diameter increases. In addition, the smaller aperture may not only cause insufficient contact surface between water and air, but it may also increase the separation resistance during oil-water separation. In conclusion, the 400 mesh filter screen was selected as the next experimental exploration.

### 3.3. Surface Structure and Super Hydrophobicity of the Filter Screen with Different Layers of Film

**Figure 6:**
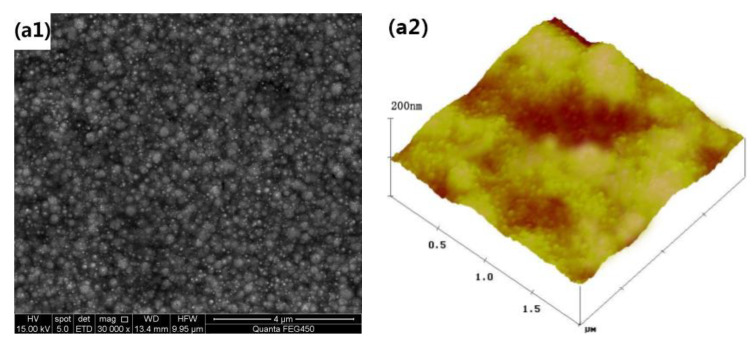

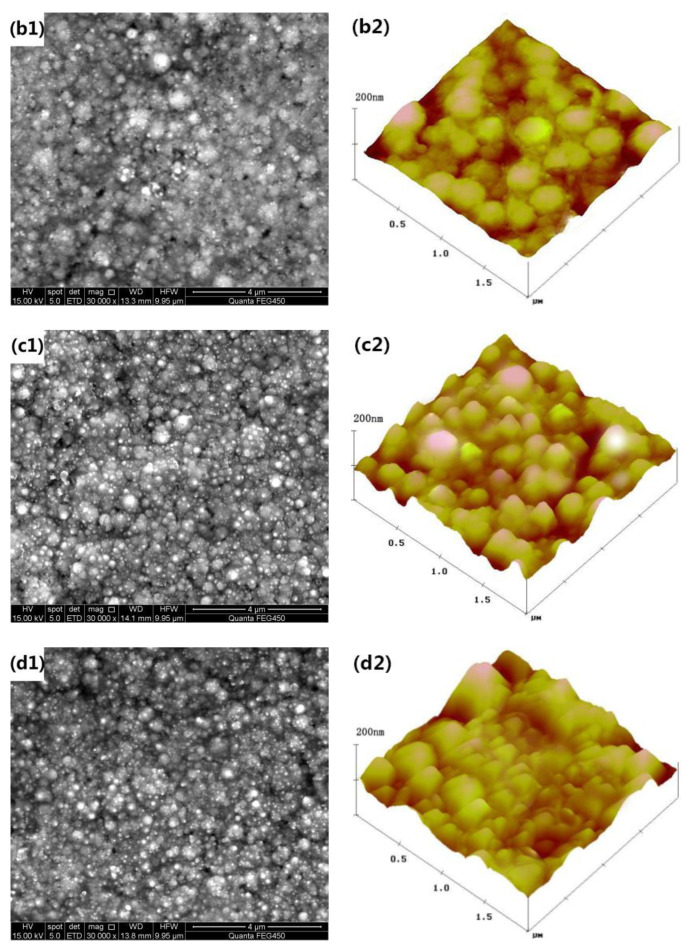
(**a1**–**d1**) SEM images of the filter film (400 meshes), which was heated and fixed by 0, 1, 2 and 3 spin-coating phenolic resin and hydrophobic nano-silica. (**a2**–**d2**) AFM image for the front side of the filter film (400 meshes), which was heated and fixed by 0, 1, 2 and 3 spin-coating phenolic resin and hydrophobic nano-silica.

In order to observe the influence of different layers of film on hydrophobicity, we used SEM and AFM to observe the surface morphology and surface roughness of the SiO_2_ film filter screen, taking the 400 mesh filter screen as an example in this paper. The test results are shown in Figure 6.

Based on the SEM and AFM in Figure 6, the surface of the original filter screen sample without spin coating is smooth (Figure 6(a1, a2)). The sample surface with a layer of spin coating has a certain nano-porous structure, which is combined with the micron mesh to form a certain micro-nano-structure. However, the appropriate rough structure cannot be constructed due to the thin coating; the contact angle is small and the rolling angle is large (Figure 6(b1, b2)). Thus, the surface is not super hydrophobic. The sample surface with two layers of spin coating film has an abundant nano-porous structure. The roughness and contact angle are substantially increased, while the rolling angle is significantly smaller. In addition, this sample has superhydrophobic properties (Figure 6(c1, c2)). Although the surface of the sample with three layers of spin coating is superhydrophobic, the surface roughness barely changes (Figure 6(d1, d2)). From the AFM diagram, we have observed that the original surface is relatively flat. When the hydrophobic SiO_2_ nanoparticles undergo continuous coating, the sample surface roughness is increased significantly. When two layers of coating are spun, the surface roughness barely changes. According to the Cassie Baxter model, the roughness coefficient increase is the key for the construction of a super hydrophobic coating.

**Figure 7:**
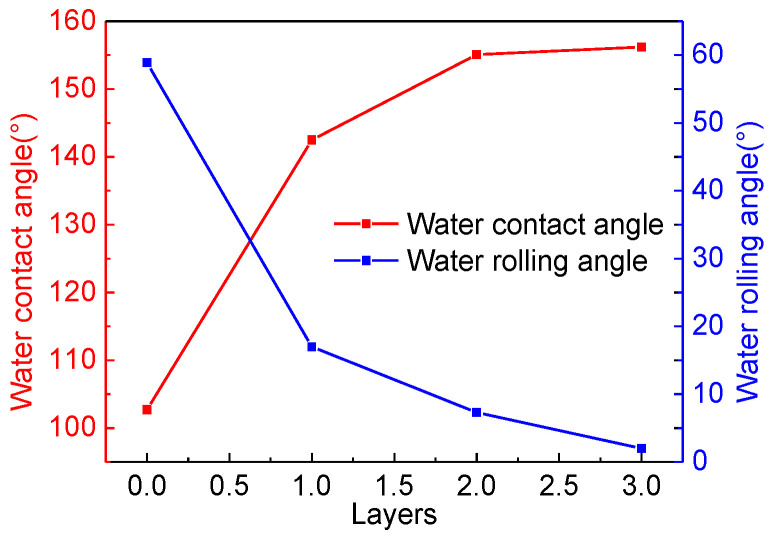
Water contact angle and water sliding angle of the filter film (400 meshes) with 0–3 layers.

As shown in Figure 7, in order to explore the impact of different film layers on the filter screen, we measured the water contact angle on the phenolic resin coating without SiO_2_ nanoparticles; the contact angle was 104.1° ± 0.3°. When a layer of SiO_2_/phenolic resin coating was applied, the contact angle of the surface water suddenly changes to 143.5° ± 0.5°, and the rolling angle decreased from 58.9° to 17.1°. When two or three layers of SiO_2_/phenolic resin coatings were applied, the contact angle of the surface water was 155.1° ± 0.2° and 156.2° ± 0.2°, respectively; no significant improvement was found. In addition, considering that the increasing number of layers will affect the efficiency of oil-water separation, the filter with two-layer SiO_2_/phenolic resin coating was finally adopted in the experiment, which not only saved the operation time of preparing the coating, but also reduced the time required for oil-water separation. To sum up, the spin coating of two-layer films was used for this experimental exploration.

### 3.4. Study on Acid, Alkali and Organic Solvent Resistance of Coated Filter Screen

**Figure 8:**
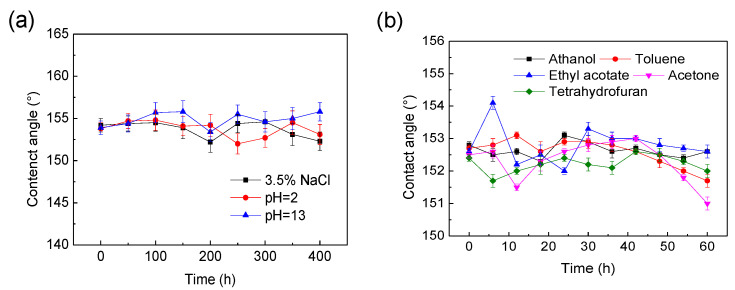
Relation between contact angle of filter mesh surface and soaking time after soaking in different chemical media: (**a**) acid–alkali–salt resistance; (**b**) organic solvent resistance.

In order to test the chemical resistance of the SiO_2_ phenolic resin-coated filter screen, different pH solutions and different oil solvents were used to test its hydrophobicity. The test results are shown in Figure 8.

The super hydrophobic and super lipophilic coating film could be damaged under harsh application conditions such as strong acid, strong base and solvent; thus, the chemical stability of the coating film has important impact on the actual application of the coating film [18]. To judge the chemical stability of the film, the filter screen was immersed into a strong acid, strong alkali, salt solution and solvent to measure its contact angle. The sodium chloride solution environment can test the influence of three corrosion factors, water, oxygen and chloride ions. Figure 8a shows the change trend of the water contact angle of the coating samples immersed in hydrochloric acid solution with pH = 2, sodium hydroxide solution with pH = 13, 3.5 wt.% sodium chloride solution, ethanol, toluene, ethyl acetate, acetone and tetrahydrofuran with time. It can be seen from Figure 8b that the water contact angle of the film surface has no obvious change during the 400 h immersion in acid, alkali, salt solution, and the organic solvent immersion. The water contact angle of the film surface keeps above 150° during 60 h in solvent. It has good acid and alkali resistance and solvent corrosion resistance. The test results showed that the prepared samples have excellent super hydrophobic and super lipophilic properties, acid and alkali resistance, solvent corrosion resistance, chemical stability, and high engineering application potentials.

### 3.5. Study on Oil–Water Separation Efficiency of the Filter Screen

**Figure 9:**
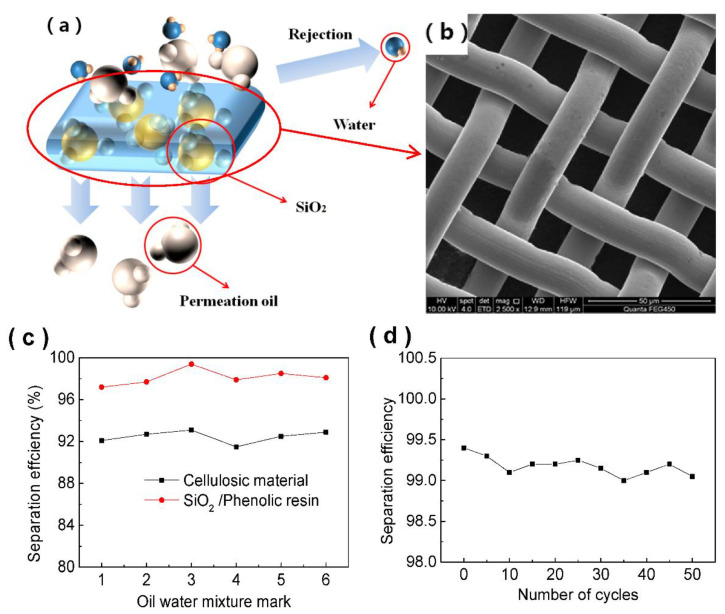
(**a**) Schematic of SiO_2_/phenolic resin for enhanced oily wastewater separation performance. (**b**) Schematic of SiO_2_/phenolic resin partial enlarged view. (**c**) Oil–water separation efficiency of different oils. Oil–water mixture mark: 1, vegetable oil; 2, methylbenzen; 3, n-hexane; 4, carbon tetrachloride; 5, dichloromethane; 6, tr-chloromethane; (**d**) oil-water separation cycles.

In order to study the oil-water separation efficiency of a SiO_2_/phenolic resin-coated filter screen, we used different kinds of oil-water mixtures to test it and conducted 60 repeated tests on the oil-water mixture with the highest separation efficiency to verify the durability of the SiO_2_/phenolic resin-coated filter screen in this paper. The test results are shown in Figure 9.

The oil-water separation principle of the SiO_2_/phenolic resin film filter screen is shown in Figure 9a. The oil-water mixture passes through the phenolic resin film filter screen with SiO_2_ particles from the top. Because of the special lipophilicity and hydrophobicity of the filter screen, the oil and water in the oil-water mixture are separated. The upper part prevents water from passing through, while the oil can smoothly pass through the filter screen to the lower part. Figure 9b is a partial enlarged view of the SiO_2_/phenolic resin-coated filter screen. Figure 9c shows a comparison between the filter screen sample and the oil-water separation efficiency of the modified fiber membrane in the literature [14] through water and different oils. In this paper, several oils such as edible oil, isooctane, gasoline, n-hexane, dodecane, dichloromethane, and trichloromethane were selected for the experiment. The oil-water separation efficiency of all oils tested reached more than 97%, and the n-hexane separation efficiency was the highest, reaching 99.4%. The lowest separation efficiency of edible oil was 97.2%, while the oil-water separation efficiency of modified fiber membrane was also positive. For hexane, the separation efficiency was the highest, about 93.1%, while the carbon tetrachloride separation efficiency was the lowest, about 91.5%. By comparing the two oil-water separation materials, it was concluded that the oil-water separation efficiency of n-hexane increased by about 6.3%. Figure 9d shows the trend chart of oil-water separation efficiency for multiple separations of the n-hexane and water mixture by the sample filter. After 50 cycles of oil-water separation, the water contact angle changed from 154.1° to 153.2°, while the separation efficiency decreased from 99.4% to 99%. The separation efficiency was still above 99% after multiple cycles. To sum up, the experimental results show that this type of filter screen had excellent super hydrophobic and super lipophilic performance, and its oil-water separation effect was also excellent and stable.

### 3.6. Analysis of Oil–Water Separation Model

**Figure 10:**
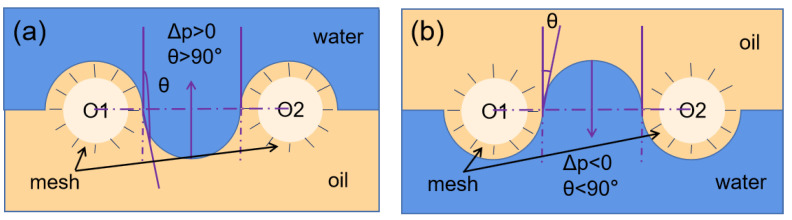
Principle of oil-water separation. (**a**) Before oil-water separation, (**b**) After oil-water separation.

In order to study the oil-water separation process, we discuss the interaction between oil and water and the filter screen in the oil-water separation process, and further explain the working mechanism of the SiO_2_/phenolic resin-coated filter screen in this paper. The oil-water separation diagram is shown in Figure 10.

To make the oil-water separation process clearer, we have drawn the oil-water separation model, as shown in Figure 10. Formula (1) is used to describe the model [19]:

(2) $$\Delta p=\frac{2\gamma }{R}=-l\gamma (\cos\theta )/A$$

In Equation (2), *γ* is the surface tension of the liquid; *l* is the perimeter of the hole; *R* is the radius of curvature; *A* is the area of the hole; *θ* is the forward contact angle of the membrane surface.

When *θ* > 90°, the film can withstand a certain pressure because ∆*p* > 0. On the contrary, when *θ* < 90°, liquid can spontaneously pass through the membrane, because ∆*p* < 0.

Figure 10a is a schematic diagram of the intermediate state of water on the membrane. This membrane contains phenolic resin material, which has a certain amount of lipophilicity. The angle *θ* is very small and ∆*p* < 0, and the membrane cannot bear any pressure. When the oil contacts the membrane, it spontaneously penetrates the membrane under the action of gravity. When water contacts the membrane, the membrane is first moistened by oil, and the multi-layer structure is occupied by oil, which enhances the water resistance of the membrane. The separation membrane maintains a super hydrophobic state *θ* greater than 90° and ∆*p* > 0, and the membrane can maintain a certain water pressure resistance within a certain maintenance range (Figure 10b). Therefore, water cannot penetrate the membrane and is trapped on the membrane. The surface free energy of the super hydrophobic and super lipophilic surfaces is between the surface tension of water (*γ* = 0.073 N/m) and the surface tension of the oil (usually *γ* = 0.020–0.030 N/m). As shown in Figure 10, the mesh is approximately cylindrical, and the super hydrophobic and filter screen is approximately a combination of many capillary structures. When the water contacts the filter screen, it takes a convex shape in the mesh. The convex shape can maintain the water under the Cassie Baxter model, making the water more mobile and excluded. When the oil contacts the filter screen, the oil can wet the mesh and present a concave shape, while the concave shape can make the oil present the Wenzel state [20] and then soak the mesh. The two curved liquid surfaces will generate a pressure pointing to water and a pressure pointing to the mesh, so that the filter screen can achieve the characteristics of oil penetration and water interception.

## 4. Conclusions

In the current research, a new filter screen was successfully designed and fabricated by heating and curing hydrophobic nano-SiO_2_/phenolic resin on the surface of a filter screen. Since the heat resistance of the hydrophobic resin filter screen is extremely strong, the coating layer fully meets the thermosetting conditions. Major conclusions based on our experimental test results are drawn as follows:

(1) A superhydrophobic and lipophilic SiO_2_/phenolic resin-coated filter screen was successfully designed and fabricated by introducing hydrophobic nano-SiO_2_ particles into the rotary coated phenolic resin through heating, baking, and curing. The surface roughness of the filter screen was enhanced substantially. As a result, the water contact angle and the hydrophobicity were increased, which greatly improved the efficiency of the oil-water separation.
(2) The hydrophobic nano-SiO_2_ concentration, the number of spinning coated SiO_2_/phenolic resin film layers, and the mesh diameter of the filter screen were analyzed deeply. These factors control the water contact angle of the SiO_2_/phenolic resin-coated filter screen. The optimized fabrication conditions were determined as: hydrophobic nano-SiO_2_ with a concentration of 30%, two layers of SiO_2_/phenolic resin film by spinning coated, and pore diameter of 400. The filter screen prepared under these conditions had a large surface roughness and good hydrophobicity.
(3) The highest oil-water separation efficiency of the fabricated SiO_2_/phenolic resin filter screen for n-hexane was 99.4%, which is about 6.3% higher than that of the fiber membrane. After 50 cycles of continuous oil-water separation, the oil-water separation efficiency for n-hexane was still above 99%, indicating that the coated filter screen has good durability. The preparation process of the filter screen was simple, and the various oil-water separation efficiencies was better.

## Figures and Tables

**Table 1:** Main experimental drugs.

Reagent Name	Reagent Specification	Origin of Reagent (China)
Hydrophobic nano-SiO_2_	HB-620	Guangzhou Jibisheng Technology Industry Co., Ltd.
Ethyl acetate	AR	Guangdong Guanghua Technology Co., Ltd.
Anhydrous ethanol, methylene blue	AR	Tianjin Damao Chemical Reagent Factory
Phenolic resin	6107	Dongguan Jincai New Materials Co., Ltd.
Stainless steel strainer	/	Guangzhou Dakun Screen Factory
Isooctane, dodecane, n-hexane, acetone	AR	Shanghai Lingfeng Chemical Reagent Co., Ltd.
10 # aviation hydraulic oil	/	Shanghai Xishun Industrial Co., Ltd.
Edible oil	/	Yihai (Guangzhou) Grain and Oil Industry Co., Ltd.
Deionized water	/	Self-control
Sudan II	/	Shenyang No. 3 Reagent Factory

**Table 2:** Main experimental equipment and instruments.

Instrument or Equipment	Model	Manufacturer (China)	Test Index
Collecting type constant temperature heating magnetic stirrer	DF-101S	Gongyi Yingyu Yuhua Instrument Factory	Rotating speed 1400 r/min
Analytical balance	FA1104	Shanghai Jingke Tianping	Weighing range 0–110 g, accuracy 0.1 mg
Bench leveler	KW-4A type	Institute of Microelectronics, Chinese Academy of Sciences	Rotating speed 3000 r/min
Intelligent digital ultrasonic instrument	KQ-500D	Dongguan Keqiao Ultrasonic Equipment Co., Ltd.	Ultrasonic power 200 W
Contact angle measuring instrument	JC200C1	Shanghai Zhongchen Digital Technology Equipment Co., Ltd.	Contact angle measurement resolution 0.01°
Electric thermostatic blast drying oven	101-1	Shanghai Experimental Instrument Factory	Temperature range 5°–250°

## Data Availability

The raw/processed data required to reproduce these findings cannot be shared at this time, as the data also form part of an ongoing study.
